# National Surveillance-Based Retrospective Longitudinal Analysis of Dementia Prevalence Trends in the Republic of Korea, 2016–2025, with a One-Year 2026 Forecast Using National Health Insurance Service Administrative Data

**DOI:** 10.3390/jcm15135122

**Published:** 2026-07-01

**Authors:** Hyeran Jung, Minsun Jung

**Affiliations:** Department of Pathology, College of Medicine, Yonsei University, 50-1 Yonsei-Ro, Seodaemun-Gu, Seoul 03722, Republic of Korea; phdgrace@yuhs.ac

**Keywords:** dementia prevalence, forecast, time-series analysis, National Health Insurance Service, population ageing, sex disparities, regional variation

## Abstract

**Background/Objectives**: Dementia poses a major public health challenge in South Korea, where population ageing has accelerated rapidly. We aimed to describe national 2016–2025 dementia prevalence trends and to generate a one-year 2026 forecast using NHIS-linked administrative data. **Methods**: We performed a retrospective longitudinal analysis of de-identified aggregate administrative data from the Ministry of Health and Welfare Municipal and District Dementia Status Report (2016–2025) and NHIS age–sex pivot table records. This was a purely descriptive and forecasting analysis; no inferential statistical tests were applied. Descriptive statistics were computed for national, sex-stratified, regional, and age-stratified outcomes. For the 2026 forecast, we used a prespecified demographic-offset time-series approach: the population aged 65 years or older and the estimated prevalence rate were modelled separately using damped-trend Holt exponential smoothing, then recombined to estimate patient count. Residual bootstrap resampling (20,000 iterations) was used to derive a 95% prediction interval for the patient count. A sensitivity analysis using ARIMA(1,1,0). **Results**: 602,633 (8.89%) in 2016 to 955,585 (9.09%) in 2025, a 58.6% increase. Females consistently showed higher prevalence than males (2025: 9.55% vs. 8.52%), and regional analysis identified South Jeolla Province (10.17%) as the highest-prevalence region and Ulsan Metropolitan City (8.10%) as the lowest. The female-to-male cumulative rate ratio reached 2.49 in the ≥85-year group. The primary 2026 forecast estimated 1,003,407 dementia patients among adults aged 65 years or older, with an estimated prevalence of 9.19% and a 95% prediction interval of 983,838–1,022,158. **Conclusions**: South Korea’s rising dementia burden is primarily driven by population ageing rather than a sharp increase in age-specific prevalence. The 2026 forecast supports urgent planning for a national dementia care population of approximately one million people.

## 1. Introduction

Dementia is one of the most consequential neurodegenerative conditions associated with global population ageing, imposing substantial burdens on affected individuals, caregivers, healthcare systems, and national economies [1]. Globally, the number of people living with dementia is projected to triple by 2050, reaching approximately 153 million, with the steepest increases expected in East Asia [2]. South Korea is among the world’s most rapidly ageing societies and formally entered super-aged society status in 2025, with adults aged 65 years or older constituting more than 20% of the total population. Several international dementia forecasting studies, including the GBD 2019 Dementia Forecasting Collaborators, have highlighted the need for country-specific longitudinal analyses to support rational policy planning [3]. In this context, providing a comprehensive national surveillance-based description of dementia burden trends and generating a near-term forecast are of direct relevance to healthcare system planning and resource allocation.

The National Health Insurance Service (NHIS) provides near-universal population coverage and maintains rich administrative records that have supported several prior dementia epidemiological studies in Korea. For example, Jang et al. (2021) [4] reported dementia prevalence and incidence using the NHIS Senior Cohort, and Hwangbo et al. (2023) [5] analysed 12-year dementia incidence and population-attributable fractions using a national longitudinal cohort. Additionally, Choi et al. [6] examined dementia prevalence using hospital utilisation data from 2008 to 2016, and Kim et al. [7] conducted an earlier nationwide survey of dementia and mild cognitive impairment prevalence. However, these prior studies either focused on earlier time periods, specific cohort subsets, or did not integrate the Ministry of Health and Welfare Municipal and District Dementia Status surveillance framework as the primary data source. No published study has simultaneously provided a unified 2016–2025 longitudinal description of national dementia burden from surveillance data and an internally consistent one-year 2026 forecast. This study addresses that gap by integrating Ministry of Health and Welfare surveillance records with NHIS age–sex pivot table data to provide both a ten-year descriptive epidemiological analysis and a prespecified near-term forecast for healthcare planning. Crucially, because the primary outcome is derived from an administrative surveillance algorithm rather than clinical diagnosis, the findings reflect estimated surveillance-based dementia burden rather than confirmed clinical prevalence.

This study had four objectives: (1) to describe national 2016–2025 dementia prevalence trends and absolute patient counts; (2) to characterise sex-stratified and age-stratified patterns; (3) to compare regional burden across the 17 administrative regions in 2025; and (4) to provide a one-year 2026 forecast grounded in recent national surveillance trends.

## 2. Methods

### 2.1. Study Design and Ethical Approval

This retrospective longitudinal study used publicly available, de-identified aggregate administrative data covering 1 January 2016, to 31 December 2025. The study was approved by the Institutional Review Board of Chungnam University, Daejeon, Republic of Korea (IRB No. 202601-SB-022-01). The requirement for informed consent was waived because no identifiable personal data were used.

### 2.2. Data Sources

Two data sources were used. The primary source was the Ministry of Health and Welfare Municipal and District Dementia Status Report (2016–2025) [8], published annually by the Korean Ministry of Health and Welfare and publicly accessible through the Korean Open Government Data Portal (data.go.kr). This report aggregates de-identified data on the older adult population (aged ≥65 years), estimated dementia patient counts, and estimated prevalence rates, stratified by sex, five-year age group (60–64, 65–69, 70–74, 75–79, 80–84, ≥85 years), and 17 administrative regions. The surveillance algorithm underlying this report uses a combined claims-based and prescription-based case definition applied to the NHIS health insurance database; specifically, individuals who received both a dementia diagnostic code (ICD-10: F00–F03, G30) and a dementia-related medication prescription within the reporting year are counted as estimated dementia cases. This case definition captures administratively ascertained rather than clinically confirmed diagnoses, and readers should interpret the prevalence figures as estimated surveillance-based indicators rather than true population prevalence derived from diagnostic evaluation. No individual-level linkage, data cleaning, or harmonisation procedures beyond the published aggregate extraction were required, as all analyses were performed on pre-aggregated annual summary tables. The secondary source was an NHIS-derived age–sex pivot table [9] summarising cumulative estimated dementia indicators across 2016–2025, extracted from NHIS aggregate outputs and used exclusively for the age-stratified analysis presented in Section 3.4. This secondary dataset is partially derived from the same underlying NHIS administrative system as the primary surveillance report but is provided in a different tabular format designed to enable age–sex cross-tabulation.

### 2.3. Study Population and Outcome Definitions

The main analytic population comprised adults aged 65 years or older. The primary outcome was estimated dementia prevalence rate, defined as the estimated number of dementia patients divided by the population aged 65 years or older. Secondary outcomes included absolute patient count, female–male prevalence gap, regional composition ratio, and age-stratified cumulative indicators.

### 2.4. Statistical Analysis

All data processing was performed using Python (version 3.11; Python Software Foundation, Wilmington, DE, USA) with pandas (version 2.2.2), matplotlib (version 3.9.0), and statsmodels (version 0.14.2). Descriptive statistics were used for observed 2016–2025 trends. Relative change was calculated as: [(2025 value − 2016 value)/2016 value] × 100%.

For the prespecified 2026 forecast, we used a demographic-offset time-series framework. Specifically, the older adult population denominator (aged ≥65 years) and the estimated prevalence rate were modelled separately and then recombined as: estimated patient count = forecast population × forecast prevalence/100. This separate-modelling approach was chosen because population growth and age-specific prevalence are driven by distinct demographic and epidemiological processes; modelling them jointly or directly forecasting patient counts would conflate these mechanisms and reduce interpretability. We explicitly acknowledge that this approach assumes a stable multiplicative relationship between population size and prevalence over the short forecast horizon; if prevalence shifts nonlinearly with age composition or if case ascertainment practices change, the recombined forecast may be affected.

Because only ten annual observations were available, we prioritised a parsimonious model to reduce overfitting and selected damped-trend Holt exponential smoothing as the primary forecasting model [10]. The damped-trend specification was preferred over simple exponential smoothing or linear Holt–Winters methods based on its ability to moderate trend extrapolation in short series, which is appropriate when a near-plateau in prevalence has been observed. Model parameters (smoothing coefficients α, β, and damping factor ϕ) were estimated by minimising the mean squared error on the in-sample one-step-ahead forecasts. Residual diagnostics confirmed approximate stationarity of residuals, with no systematic autocorrelation detected at lag 1. Because the short series precludes formal out-of-sample back-testing, forecast accuracy was assessed by leave-one-out cross-validation on the available observations; the mean absolute percentage error across these assessments was approximately 0.4% for population and 0.3% for prevalence. Residual bootstrap resampling with 20,000 iterations was used to derive the 95% prediction interval for the recombined patient-count forecast. The sensitivity analysis used ARIMA(1,1,0) models fit separately to the population and prevalence series; the ARIMA specification was selected based on autocorrelation and partial autocorrelation function inspection and the Akaike information criterion. All analyses were purely descriptive and forecasting-based; no inferential statistical tests were applied. The study is, therefore, presented as a descriptive epidemiological and time-series forecasting analysis, and language implying statistical significance should not be inferred from the results. During the preparation of this manuscript, the author(s) used Claude (Anthropic, San Francisco, CA, USA) to assist with English-language editing and manuscript formatting during the revision process; no analytic code, statistical results, or data interpretation was generated by the tool. The authors reviewed and verified all AI-assisted edits and take full responsibility for the content of this publication.

## 3. Results

### 3.1. National Longitudinal Trends in Dementia Burden, 2016–2025

**Figure 1:**
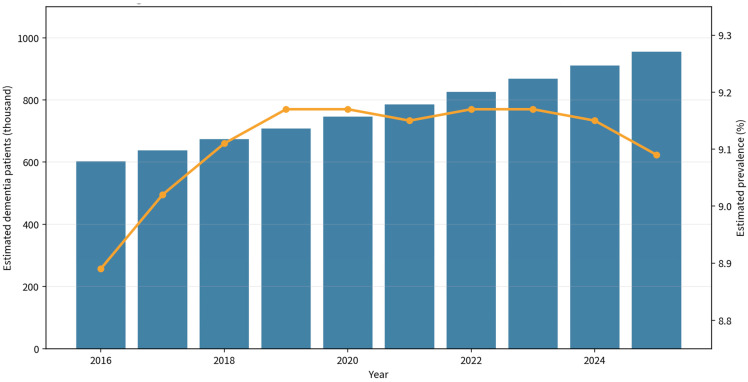
National trends in estimated dementia burden, South Korea, 2016–2025. Bars show estimated dementia patient counts (left axis) and the overlaid line shows annual estimated prevalence (right axis). The plateau in estimated prevalence near 8.9–9.2% since 2019 should not be interpreted as indicating a stable underlying disease risk; this pattern could also reflect changes in administrative ascertainment, diagnostic coding practices, prescription patterns, survival, or access to care over the observation period.

The estimated number of dementia patients among adults aged 65 years or older increased from 602,633 in 2016 to 955,585 in 2025, corresponding to a 58.6% relative increase. During the same period, the older adult population increased from 6,781,159 to 10,508,726 (+55.0%). In contrast, the estimated prevalence rate remained comparatively stable, ranging from 8.89% to 9.17%. This pattern indicates that the growing burden of dementia was mainly driven by the expansion of the older population (Table 1; Figure 1).

### 3.2. Sex-Stratified Analysis

**Figure 2:**
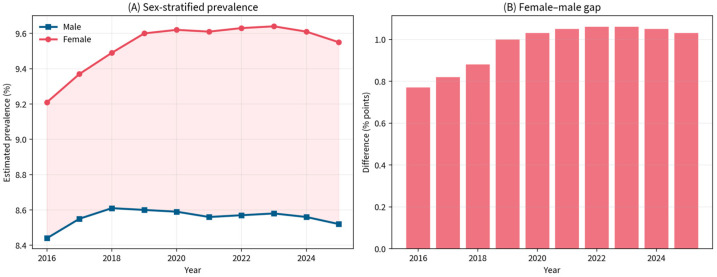
Sex-stratified estimated dementia prevalence, South Korea, 2016–2025. (**A**) shows annual estimated prevalence (%) by sex; (**B**) shows the female–male prevalence gap (percentage points). All values are descriptive surveillance summaries derived from administrative data and do not represent inferential statistical comparisons. Minor year-to-year fluctuations in the sex gap reflect surveillance variability rather than confirmed epidemiological change.

Female estimated dementia prevalence exceeded male prevalence in every study year. In 2025, female prevalence was 9.55% compared with 8.52% in males, yielding a female–male gap of +1.03 percentage points. The gap widened from +0.77 percentage points in 2016, consistent with the progressive concentration of dementia burden among older women (Table 2; Figure 2).

### 3.3. Regional Analysis, 2025

**Figure 3:**
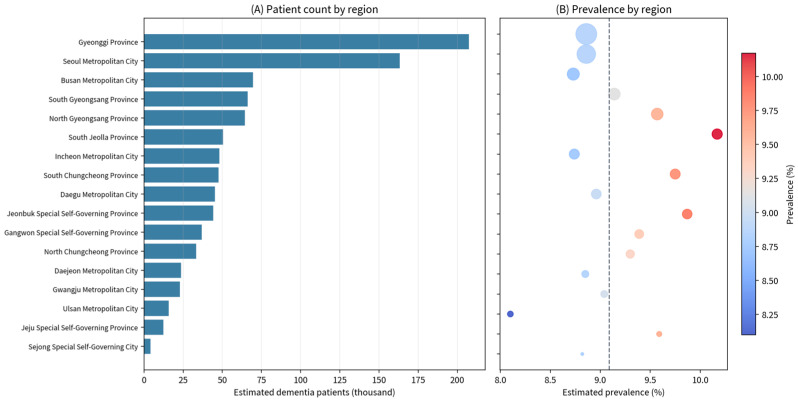
Regional distribution of estimated dementia burden, South Korea, 2025. (**A**) shows estimated patient counts by region (absolute burden; reflects both population size and prevalence). (**B**) shows estimated prevalence rate (%) by region, with bubble size proportional to absolute patient count. High absolute burden and high prevalence are distinct constructs: (**A**) is dominated by large-population metropolitan regions, whereas (**B**) reflects age-specific disease concentration independent of regional population size. Age standardisation was not applied; regional prevalence differences may partly reflect differences in age structure rather than differences in underlying disease risk.

The three highest-burden regions by absolute patient count in 2025 were Gyeonggi Province (207,395), Seoul Metropolitan City (163,213), and Busan Metropolitan City (69,629), together accounting for 46.1% of all national estimated patients. By estimated prevalence, the highest-burden regions were South Jeolla Province (10.17%), Jeonbuk Special Self-Governing Province (9.87%), and South Chungcheong Province (9.75%), whereas Ulsan Metropolitan City showed the lowest estimated prevalence (8.10%) (Table 3; Figure 3). Readers should note that age standardisation was not applied to these regional comparisons; observed differences in estimated prevalence across regions may partly reflect differences in regional age structure rather than differences in age-specific dementia risk. High absolute patient count and high estimated prevalence are distinct constructs: regions with high absolute counts are primarily large metropolitan areas, whereas regions with high estimated prevalence are largely rural provinces, which tend to have older age compositions.

### 3.4. Age-Stratified Analysis from NHIS Pivot Table Data, 2016–2025

**Figure 4:**
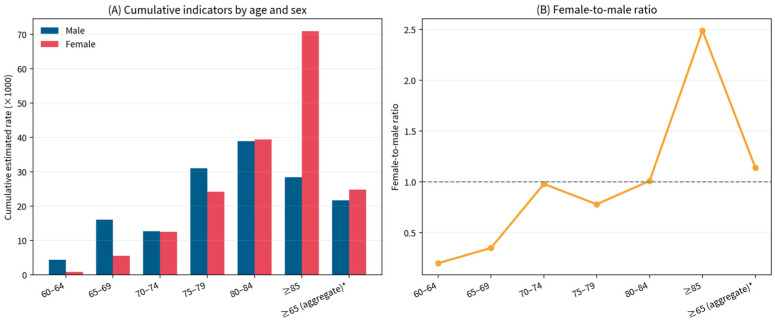
Age-stratified cumulative estimated dementia indicators by sex, South Korea, 2016–2025. (**A**) shows the sum of annual estimated prevalence rates (“percentage-years”) across 2016–2025, by age group and sex; these are not annual prevalence rates but ten-year cumulative aggregates. The asterisk (*) denotes the ‘≥65 (aggregate)’ bar, which is derived directly from the overall (non-age-stratified) NHIS pivot table summary for the ≥65-year population and is not the arithmetic sum of the six preceding age-specific bars. (**B**) shows the female-to-male cumulative rate ratio by age group. The ratio exceeds 2.0 in the ≥85-year group because it reflects cumulative ten-year concentration of burden rather than contemporaneous annual prevalence; this should not be interpreted as a two-fold difference in annual dementia rate between sexes in this age group.

Age-stratified NHIS indicators showed a steep gradient in estimated dementia burden across age groups, with the female-to-male cumulative rate ratio reaching 2.49 in the ≥85-year group. This cumulative indicator represents the sum of estimated annual prevalence rates across 2016–2025 (“percentage-years”), not an annual prevalence rate; therefore, the ratio of 2.49 reflects the ten-year cumulative concentration of burden among very old women relative to very old men and should not be interpreted as a two-fold annual prevalence difference. This pattern is consistent with the known demographic concentration of the very oldest population among women and highlights the combined effect of differential longevity and older-age accumulation on surveillance-based dementia burden (Table 4; Figure 4).

### 3.5. 2026 Forecast of National Dementia Burden

**Figure 5:**
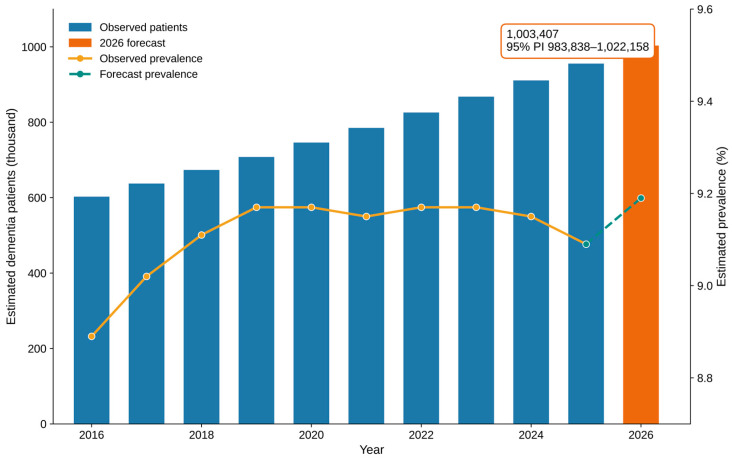
One-year forecast of national dementia burden, South Korea, 2026. Observed 2016–2025 values are shown with the model-based 2026 forecast. The point estimate is 1,003,407 estimated patients at a prevalence of 9.19%, with a 95% prediction interval of 983,838–1,022,158.

The primary time-series model forecasted a 2026 population aged 65 years or older of 10,914,917, an estimated dementia prevalence of 9.19%, and 1,003,407 estimated dementia patients (95% prediction interval [PI] for patient count: 983,838–1,022,158). The forecasted prevalence of 9.19% represents a modest increase from the 2025 observed value of 9.09%, consistent with the slight upward inflection modelled by the damped-trend Holt smoothing on the prevalence series. The sensitivity ARIMA-based forecast yielded 953,081 patients with an estimated prevalence of 9.04%; a formal prediction interval for the sensitivity patient-count forecast was not computed as this was a secondary analysis. Together, the two approaches define a plausible planning range of approximately 953,000 to 1,023,000 estimated dementia patients in 2026. These projections suggest that South Korea is likely to cross or approach the threshold of approximately one million estimated dementia patients in 2026 while maintaining an estimated prevalence level near the observed 9% range (Table 5; Figure 5). Given the reliance on only ten observations and the short-series limitations described below, these forecasts should be interpreted as near-term planning estimates rather than definitive predictions.

## 4. Discussion

This longitudinal analysis provides four principal findings. First, national dementia burden increased substantially in absolute terms from 2016 to 2025 (+58.6%), but the estimated prevalence rate remained comparatively stable near 9%. Second, females had consistently higher estimated prevalence than males, and the female–male gap widened modestly over time. Third, regional disparities followed a dual pattern in which metropolitan regions carried the greatest absolute burden while rural provinces showed the highest prevalence rates. Fourth, the 2026 time-series forecast indicates that South Korea is likely to face an estimated dementia care population approaching one million older adults in 2026. Taken together, these findings characterise a period in which demographic expansion—rather than rising age-specific risk—has been the dominant driver of growing national dementia burden, consistent with the dissociation between absolute counts and prevalence rates observed internationally.

The forecast results extend the descriptive findings into a near-term planning horizon. The GBD 2019 Dementia Forecasting Collaborators [3] projected substantial absolute increases in dementia cases across East Asia driven primarily by demographic ageing, a pattern closely mirrored by the present findings. Comparable national surveillance analyses in Japan and Taiwan have likewise documented stable prevalence alongside rising absolute counts [11,12,13], suggesting that the observed Korean pattern is consistent with regional demographic trends. The 2026 primary forecast of approximately 1,003,407 estimated patients (95% PI 983,838–1,022,158) represents a policy-relevant threshold that supports immediate planning for dementia care capacity, consistent with the priorities outlined in Korea’s 4th National Dementia Plan [14]. These policy priorities should include expansion of dementia care centres, diagnostic services, home-based care, caregiver support, and long-term care insurance capacity, as well as age- and sex-targeted prevention strategies targeting the known modifiable risk factors identified in the Lancet Commission 2020 [15] report, and supported by evidence on the projected benefit of risk-factor reduction and multidomain prevention trials [16,17]. The narrower sensitivity forecast (953,081 patients) provides important context: the two estimates define a plausible planning range of approximately 953,000 to 1,023,000 estimated patients, and policy preparation should be designed to accommodate the upper end of this range. Notably, the observation that prevalence has plateaued near 9% since 2019 does not necessarily indicate a stable underlying disease risk; it could also reflect changes in administrative ascertainment practices, diagnostic coding, drug prescription patterns, or survival. This interpretive caution is important when using the forecast to infer future clinical burden.

The persistent female excess in estimated dementia prevalence is consistent with global epidemiological findings. Ferretti et al., Mielke et al., and Beam et al. [18,19,20] have documented higher female dementia prevalence in multiple international cohorts, attributing this pattern to a combination of differential longevity, population age structure, hormonal factors, and social determinants. The widening female–male gap observed in this study (from +0.77 percentage points in 2016 to +1.03 in 2025) is consistent with the progressive concentration of the very old population—those aged 85 years or older—among women. The cumulative ten-year female-to-male ratio of 2.49 in the ≥85 age group reflects this demographic concentration; readers should note this is a cumulative surveillance indicator, not an annual sex-specific prevalence ratio. These findings support sex-targeted screening and care coordination strategies, particularly for very old women. However, because the analytic design is purely descriptive and ecological, no causal inference regarding the biological or social mechanisms underlying sex differences can be drawn from this dataset.

Regional findings also have direct planning implications but require careful interpretation. The most important interpretive constraint is that age standardisation was not applied to regional prevalence estimates; this is a central analytical limitation rather than a minor caveat. Because age structures differ substantially across Korean administrative regions—with rural provinces generally having older age compositions than metropolitan cities—the observed higher estimated prevalence in South Jeolla Province (10.17%) and Jeonbuk Special Self-Governing Province (9.87%) compared with Ulsan Metropolitan City (8.10%) may partly or largely reflect differences in age structure rather than true differences in age-specific dementia risk. Consequently, the Discussion avoids strong causal interpretations of regional disparities and instead frames regional differences as descriptive surveillance patterns requiring further investigation with age-standardised methods. Notwithstanding this limitation, the patterns do have practical planning relevance: metropolitan regions require higher absolute service capacity because of larger patient volume, while rural provinces may require tailored strategies for access to early detection and long-term care. The speculative explanations regarding healthcare access, screening practices, or prevention strategies that might contribute to regional differences cannot be supported by the present ecological analysis and would require regional-level socioeconomic and healthcare utilisation data for substantiation.

## 5. Limitations

This study has several important limitations that should be considered when interpreting the findings. First, the Ministry of Health and Welfare surveillance system is based on an administrative algorithm combining claims and prescription records (ICD-10 dementia codes plus dementia-related medication prescriptions); confirmed clinical diagnoses were not used. Accordingly, all outcomes are estimated surveillance-based indicators rather than true clinical prevalence, and some degree of both false-positive and false-negative ascertainment is expected. Changes in clinical coding practices, drug prescription patterns, or surveillance algorithm definitions over the observation period could contribute to observed trends in prevalence independent of true epidemiological change. Second, the main outcome is prevalence rather than incidence, which limits the ability to distinguish between changes in new disease occurrence and changes in survival or case-finding. Third, the analytic design is ecological and purely descriptive; no inferential statistical analyses were applied, and no causal relationships between any exposure and dementia outcomes can be established. Fourth, the 2026 forecast is model-based and relies on only ten annual observations, substantially limiting robustness and precluding meaningful external validation. Short-series forecasting is inherently fragile to structural breaks, outliers, and changes in underlying trends; readers should treat the 2026 estimate as a short-term planning approximation rather than a definitive prediction. Possible structural breaks include changes in the ageing speed of the Korean population, revisions to the surveillance algorithm, or shifts in policy affecting dementia diagnosis rates. Fifth, age standardisation was not applied to regional prevalence comparisons; because regional age structures likely differ substantially, observed regional differences in estimated prevalence should be interpreted with caution and may not reflect genuine differences in age-specific dementia risk. Sixth, the sensitivity forecast (ARIMA-based, 953,081 patients) is presented without a comparable prediction interval to the primary forecast, which limits the quantitative comparison of uncertainty between the two approaches. Seventh, because no individual-level data were used and no causal modelling was performed, this study cannot examine associations between health screening indicators, risk factors, or healthcare utilisation and dementia outcomes.

## 6. Conclusions

Estimated dementia burden in South Korea increased markedly from 2016 to 2025 (+58.6% in absolute patient count), whereas estimated prevalence remained comparatively stable near 9%, indicating that demographic expansion rather than rising age-specific risk has been the primary driver of growing national burden. Females and older age groups—especially women aged 85 years or older—bore disproportionate surveillance burden, and rural provinces showed higher estimated prevalence despite lower absolute patient counts, though age-standardised analyses are needed to confirm genuine regional differences in disease risk. The 2026 forecast of approximately 1,003,407 estimated dementia patients (95% PI 983,838–1,022,158) reinforces the need for immediate policy preparation as South Korea approaches a national dementia care population of one million. Priorities should include expanded long-term care insurance capacity, community-based care services, caregiver support systems, and regionally differentiated early detection strategies. The rapidly increasing absolute patient burden also implies growing economic costs for both the public healthcare system and family caregivers, underscoring the urgency of scalable, cost-effective dementia care planning. Future research should apply age-standardised methods to regional analyses, extend the forecast horizon with updated data as it becomes available, and investigate the modifiable risk factors identified in the Lancet 2020 Commission report [15] at the national and regional level.

## Figures and Tables

**Table 1:** National trends in estimated dementia patient count and estimated prevalence among adults aged ≥65 years in South Korea, 2016–2025.

Year	Older Adult Population (≥65 y), *n*	Estimated Dementia Patients, *n*	Estimated Prevalence, %	Year-on-Year Change, % Points
2016	6,781,159	602,633	8.89	—
2017	7,066,201	637,537	9.02	+0.13
2018	7,389,480	673,532	9.11	+0.09
2019	7,718,616	708,027	9.17	+0.06
2020	8,134,674	746,102	9.17	+0.00
2021	8,577,830	785,089	9.15	−0.02
2022	9,010,545	825,857	9.17	+0.02
2023	9,462,270	867,803	9.17	+0.00
2024	9,955,476	910,898	9.15	−0.02
2025	10,508,726	955,585	9.09	−0.06

Year-on-year change denotes the arithmetic difference in estimated prevalence between consecutive years.

**Table 2:** Sex-stratified estimated dementia patient count and prevalence among adults aged ≥65 years, South Korea, 2016–2025 (selected years).

Year	Sex	Older Adult Population (≥65 y), *n*	Estimated Dementia Patients, *n*	Estimated Prevalence, %	Sex Gap (F–M), % Points
2016	M	2,858,259	241,204	8.44	+0.77
2016	F	3,922,900	361,429	9.21	
2018	M	3,153,847	271,586	8.61	+0.88
2018	F	4,235,627	401,947	9.49	
2020	M	3,515,954	301,989	8.59	+1.03
2020	F	4,618,720	444,113	9.62	
2022	M	3,942,267	337,795	8.57	+1.06
2022	F	5,068,279	488,062	9.63	
2024	M	4,406,646	377,385	8.56	+1.05
2024	F	5,548,830	533,513	9.61	
2025	M	4,674,392	398,190	8.52	+1.03
2025	F	5,834,333	557,395	9.55	

F = female; M = male. The sex gap (F−M, percentage points) represents the arithmetic difference between female and male estimated prevalence for the same year. For ease of reading, this value is displayed on the male row of each selected year; the corresponding female row shows a blank cell. Readers should note that these are descriptive surveillance summaries rather than inferential comparisons, and minor year-to-year fluctuations in the sex gap should not be over-interpreted.

**Table 3:** Regional estimated dementia patient count, prevalence rate, and national composition among adults aged ≥65 years, South Korea, 2025.

Region	Older Adult Population (≥65 y), *n*	Estimated Dementia Patients, *n*	Estimated Prevalence, %	National Composition, %
Gyeonggi Province	2,340,632	207,395	8.86	21.7
Seoul Metropolitan City	1,842,720	163,213	8.86	17.1
Busan Metropolitan City	797,382	69,629	8.73	7.3
South Gyeongsang Province	723,422	66,135	9.14	6.9
North Gyeongsang Province	672,664	64,399	9.57	6.7
South Jeolla Province	495,810	50,425	10.17	5.3
Incheon Metropolitan City	551,748	48,212	8.74	5.0
South Chungcheong Province	487,216	47,528	9.75	5.0
Daegu Metropolitan City	505,305	45,287	8.96	4.7
Jeonbuk Special Self-Governing Province	448,262	44,234	9.87	4.6
Gangwon Special Self-Governing Province	393,826	36,968	9.39	3.9
North Chungcheong Province	358,014	33,286	9.30	3.5
Daejeon Metropolitan City	266,696	23,595	8.85	2.5
Gwangju Metropolitan City	253,623	22,935	9.04	2.4
Ulsan Metropolitan City	195,456	15,835	8.10	1.7
Jeju Special Self-Governing Province	129,360	12,401	9.59	1.3
Sejong Special Self-Governing City	46,588	4109	8.82	0.4

Regions are sorted by estimated patient count in descending order.

**Table 4:** Age-stratified cumulative estimated dementia indicators by sex, South Korea, 2016–2025 (NHIS pivot table data).

Age Group (Years)	Male Cumulative Estimated Rate (2016–2025)	Female Cumulative Estimated Rate (2016–2025)	Female-to-Male Ratio
60–64	4408.80	876.75	0.20
65–69	16,082.10	5561.10	0.35
70–74	12,700.35	12,499.95	0.98
75–79	30,986.85	24,248.40	0.78
80–84	38,927.70	39,453.75	1.01
≥85	28,456.80	70,991.70	2.49
≥65 (aggregate) *	21,675.10	24,776.51	1.14

Cumulative estimated rates represent the arithmetic sum of the annual estimated dementia prevalence rate (%) within each age–sex stratum across all ten observation years (2016–2025), derived from NHIS aggregate pivot table data. The unit is, therefore, “percentage-years” (i.e., the sum of ten annual percentages), not an annual prevalence rate; these values cannot be interpreted as a prevalence rate for any single year. This cumulative indicator was used to characterise the aggregate surveillance burden over the observation decade and to compare the relative concentration of dementia burden across age and sex groups. Readers should note that year-specific age-stratified prevalence rates would provide a more conventional epidemiological summary; the cumulative indicator is presented here because year-specific age–sex–stratified values were only available in the NHIS pivot table in cumulative format. The female-to-male ratio in the ≥85-year group (2.49) reflects the cumulative concentration of burden among older women over ten years and should not be interpreted as an annual sex ratio. * The ≥65 (aggregate) row is derived directly from the overall (non-age-stratified) NHIS pivot table summary for the ≥65-year population and is not the arithmetic sum of the six preceding age-specific rows.

**Table 5:** One-year forecast of national dementia burden among adults aged ≥65 years in South Korea, 2026.

Estimate	Older Adult Population (≥65 y), *n*	Estimated Prevalence, %	Estimated Dementia Patients, *n*	Method
Observed 2025	10,508,726	9.09	955,585	Observed surveillance statistic
Forecast 2026 (primary)	10,914,917	9.19	1,003,407 (95% PI 983,838–1,022,158)	Damped-trend Holt demographic-offset model
Forecast 2026 (sensitivity)	10,547,760	9.04	953,081	ARIMA(1,1,0) demographic-offset model

PI = prediction interval. The 2026 values are model-based forecasts, not observed surveillance statistics.

## Data Availability

The datasets supporting the conclusions of this article are available from the corresponding author upon reasonable request. The primary data source is publicly available from the Ministry of Health and Welfare; NHIS data are available to approved researchers through NHIS.
